# Implant-Supported Oral Rehabilitation in Head and Neck Cancer Patients: A 20-Year Single-Center Study (2005–2024)

**DOI:** 10.3390/jcm14155435

**Published:** 2025-08-01

**Authors:** Manuel Tousidonis, Santiago Ochandiano, Carlos Navarro-Cuellar, Carlos Navarro-Vila, Javier López de Atalaya, Cristina Maza, Ana María Lopez Lopez, Ignacio Navarro-Cuellar, Alba García Sevilla, Gema Arenas de Frutos, Raul Antunez-Conde, Paloma Planells del Pozo, Jose Ignacio Salmeron

**Affiliations:** 1 Department of Oral and Maxillofacial Surgery, Hospital General Universitario Gregorio Marañón, 28007 Madrid, Spain; sochandiano@hotmail.com (S.O.); atalaya-la@hotmail.com (J.L.d.A.); nnavcu@hotmail.com (I.N.-C.); gema.arenas@gmail.com (G.A.d.F.); antunezconde_92@hotmail.com (R.A.-C.); joseignacio.salmeron@salud.madrid.org (J.I.S.); 2 Instituto de Investigación Sanitaria Gregorio Marañón (IISGM), 28007 Madrid, Spain; 3 Department of Surgery, Faculty of Medicine, Universidad Complutense de Madrid (UCM), 28040 Madrid, Spain; 4 Department of Oral and Maxillofacial Surgery, Hospital Universitario Ramón y Cajal, Instituto Ramón y Cajal de Investigación Sanitaria (IRYCIS), Ctra. de Colmenar Viejo, km 9.100, 28034 Madrid, Spain; albagsevilla@gmail.com; 5 Faculty of Dentistry, Universidad Complutense de Madrid (UCM), 28040 Madrid, Spain

**Keywords:** dental implants, oral cancer, head and neck oncology, osseointegration, radiotherapy, free fibula flap, prosthetic rehabilitation, implant survival, implant stability quotient, point-of-care manufacturing, 3D printing

## Abstract

**Background/Objectives:** Oral cancer resection often leads to maxillofacial defects and dentition loss, compromising patients’ quality of life. Implant-supported prosthetic rehabilitation offers a reliable solution to restore function, though factors such as bone reconstruction, radiotherapy, and timing of implant placement (immediate vs. delayed) may influence outcomes. This study aimed to evaluate long-term implant survival and rehabilitation timelines in oncologic patients, comparing two cohorts (2005–2014 and 2015–2024) to assess the impact of evolving clinical practices. **Methods:** A retrospective cohort study was conducted at Hospital General Universitario Gregorio Marañón (Madrid, Spain), including 304 patients who underwent ablative oral cancer surgery and subsequent implant-based rehabilitation between 2005 and 2024. Data on demographics, oncologic treatment, reconstruction, implant timing, and prosthetic rehabilitation were collected. Outcomes were compared using Kaplan–Meier analysis and appropriate statistical tests between the 2005–2014 (*n* = 122) and 2015–2024 (*n* = 182) cohorts. **Results:** A total of 2341 Ticare Implants^®^ were placed, supporting 281 prostheses. Implant placement during primary surgery increased from 41% to 71% (*p* < 0.001). The median time from surgery to prosthesis significantly decreased from 24 to 15 months (*p* < 0.001). Five-year implant survival was 95% in the early cohort versus 97% in the later cohort. Implant survival was comparable between irradiated and non-irradiated patients (~94–96%). Fixed prostheses became more frequent (92% vs. 79%, *p* = 0.002). **Conclusions:** Implant-supported rehabilitation in oncologic patients is highly feasible and durable, with improved timelines and functional outcomes associated with early implant placement and modern digital planning strategies.

## 1. Introduction

Resection of malignant tumors in the oral cavity and jaws often results in substantial hard and soft tissue defects, leading to difficulties in speaking, chewing, and swallowing. Restoring oral function in head and neck cancer survivors is therefore a critical challenge for the surgical oncology and dental teams [1]. Worldwide, head and neck cancers affect over 650,000 people annually and cause roughly 330,000 deaths each year [2]. Among these, oral squamous cell carcinoma is a major subset. Advances in oncologic therapy over past decades have modestly improved 5-year survival rates for oral cancer (from ~55% in the 1990s to ~66% in the 2000s) [3], resulting in a growing population of patients requiring post-treatment rehabilitation. Restoring both function and aesthetics through dental rehabilitation is a key determinant of post-treatment quality of life in oral cancer survivors [4,5,6]. Conventional removable dentures are often insufficient in patients with large tumor-related defects or xerostomia after radiotherapy. In contrast, implant-supported dental prostheses can dramatically improve oral function and patient satisfaction [7]. Indeed, dental implants have “revolutionized” rehabilitation of oncologic patients [8], enabling fixed prosthetic solutions even after jaw resection and reconstruction. A previous single-institution study from our center, covering the 2006–2015 period, hypothesized that implant-supported or implant-retained prosthetic rehabilitation in oral cancer patients could achieve success rates comparable to those in non-oncologic populations [9]. The results supported this assumption, showing high implant survival (~95% at 5 years), including in irradiated patients, and comparable outcomes in vascularized bone grafts and native bone. However, the cohort was relatively limited (122 patients, 941 implants) and reflected earlier surgical and prosthetic protocols.

Several factors have been postulated to influence the outcomes of dental implants in head and neck cancer patients. One primary consideration is radiotherapy, which can compromise bone vascularity and healing capacity. Some studies and meta-analyses have found that implants in irradiated jaws have lower survival rates (e.g., ~84–92%) compared to non-irradiated cases [10], and that radiation is a significant risk factor for implant failure [11]. For instance, a 2016 meta-analysis by Chrcanovic et al. reported implant survival of ~84% in irradiated bone vs. 95% in non-irradiated bone [12]. Similarly, a 2021 systematic review found ~91.9% survival in irradiated patients vs. 97% in non-irradiated after ~3 years follow-up [13]. Nevertheless, other studies have shown more optimistic results, with carefully selected irradiated patients achieving implant success comparable to non-irradiated controls [14,15,16,17,18,19,20]. The variation in the literature suggests that factors such as radiation dose to the implant site, time elapsed after radiation, use of hyperbaric oxygen, and surgical technique can modulate outcomes [20,21,22,23]. In our previous series, no significant difference in 5-year implant survival was observed between irradiated and non-irradiated patients (95% vs. 97%, *p* > 0.3), consistent with reports indicating that implant-based rehabilitation can achieve reliable outcomes in irradiated patients when appropriate patient selection and clinical protocols are applied [9].

Another key factor is the timing of implant placement relative to oncologic surgery. Traditionally, implants have been placed in a delayed fashion (secondary implants), after completion of cancer therapy and adequate healing, to avoid interfering with oncologic follow-up and to ensure patients can tolerate additional procedures. A common clinical practice has been to delay dental implant placement for approximately 18–24 months following surgery and radiotherapy, in order to allow for oncologic surveillance and to exclude early tumor recurrence prior to initiating prosthetic rehabilitation [24,25]. Taylor and Worthington recommended a minimum 2-year disease-free interval prior to implant placement in oral cancer patients [26], and in our institution, secondary implants were historically placed around 24 months post-tumor resection to maximize oncologic safety [27]. In recent years, however, there has been increasing interest in primary or immediate implant placement, performed at the same time as the tumor resection and jaw reconstruction surgery [28]. Primary placement has potential advantages: the implants can be inserted into the fresh wound (or into the bone flap outside the body before transplantation), potentially before radiotherapy is delivered, and this approach can facilitate earlier prosthetic rehabilitation [29,30]. Multiple studies have suggested higher or at least equivalent implant survival rates with primary placement compared to delayed placement [31,32], and a meta-analysis confirmed a slightly better survival rate for implants placed during ablative surgery [33]. Moreover, primary implants allow the dental rehabilitation phase to begin sooner (once healing is complete), shortening the edentulous period for the patient. On the other hand, not all patients are candidates for primary implantation (e.g., if the surgical field is heavily contaminated or if prognosis is very poor), and implant placement adds time to an already lengthy surgery. Some authors have reported no significant difference between immediate and delayed implants in this context [34], so the optimal approach may depend on individual case factors.

Additionally, the quality of the host bone (native mandible/maxilla vs. grafted bone) and the region of implant placement (anterior vs. posterior) have been considered relevant. Implants in vascularized free bone flaps (such as fibula or iliac crest) have shown excellent short-term survival (~97–98% at 1 year) comparable to native bone implants [35,36]. Our previous analysis found that implants placed in fibula or iliac crest flap bone had outcomes equivalent to those in native jaw bone, which is consistent with the literature indicating that the nature of the bone (grafted vs. native) is not a major risk factor for implant failure when vascularized grafts are used [37]. However, the location within the jaws might influence results: posterior regions (molars) often have poorer bone quality or higher load, and some studies (including our earlier data) have noted higher failure rates in posterior implants and in the mandible compared to anterior regions and maxilla. This contrasts with some systematic reviews of irradiated cases that noted maxillary implants faring slightly worse than mandibular [38]. The discrepancy could be due to our cohort’s specifics: many mandibular cases involved reconstructed segments and radiotherapy, which could compound risk in that site.

Since 2015, various technical innovations have been adopted at our center, including advanced 3D planning and printing of surgical guides for implant placement [39,40,41] and a multidisciplinary approach to optimize implant positioning (prosthetically guided surgery). In addition, the use of the Osstell^®^ system to assess implant stability through the Implant Stability Quotient (ISQ) has been incorporated as an objective tool to guide loading decisions and monitor primary and secondary stability. These developments aimed to reduce the number of non-utilizable implants—those that successfully osseointegrate but cannot be incorporated into the final prosthesis due to suboptimal positioning—commonly referred to as “sleepers”. Optimizing implant positioning and stability assessment could contribute to a more efficient rehabilitation process and improve the predictability of prosthetic outcomes. In our initial 2006–2015 series, careful planning of secondary implants yielded only 1.7% of integrated implants that ended up not supporting. With computer-aided design and intraoperative navigation or guides, we anticipated this figure could be driven even lower in recent years, maximizing the functional use of every implant placed.

Given these considerations, we expanded our retrospective analysis to include patients treated up through 2024, roughly doubling the sample size and incorporating an additional 10 years of experience. The goals of this study were (1) to evaluate the long-term success of implant-supported oral rehabilitation in a large cohort of oral cancer patients, including implant survival rates at 2 and 5 years; (2) to compare outcomes between an earlier cohort (2005–2014) and a later cohort (2015–2024) to determine if there have been improvements in implant survival, prosthetic success, or treatment timelines; and (3) to assess the impact of factors such as radiotherapy, implant timing, jaw location, and reconstructive technique on outcomes across the two periods. We hypothesized that refinements in technique and patient management in the latter period have led to slightly improved implant survival and a substantially reduced time to rehabilitation for these patients. The findings of this study are expected to inform best practices for optimizing dental implant rehabilitation in head and neck oncology patients.

## 2. Materials and Methods

### 2.1. Study Design and Patient Selection

This study is a retrospective observational analysis of oral oncologic patients who received dental implant rehabilitation at a single tertiary care center (Hospital General Universitario Gregorio Marañón, Madrid). We reviewed the records of patients treated by the Oral and Maxillofacial Surgery Department between January 2005 and December 2024. Inclusion criteria were (1) diagnosis of malignant tumor in the oral cavity or oromandibular region (predominantly squamous cell carcinoma of the mandible, floor of mouth, tongue, or maxilla) requiring ablative surgery; (2) treatment with curative intent surgery, with or without adjuvant radiotherapy; (3) placement of endosseous dental implants either simultaneously during the tumor resection surgery (primary implants) or in a secondary procedure after completion of oncologic therapy; and (4) attempt at prosthetic dental rehabilitation using the implants. Patients who did not have any implants placed (for example, those who could not undergo dental rehabilitation due to early tumor recurrence or very poor prognosis) were excluded. However, patients who had implants placed but never received a definitive prosthesis (due to death, complications, or refusal) were included in the analysis of implant integration (since implants had been inserted) but were counted as not achieving prosthetic rehabilitation.

A total of 304 patients met the inclusion criteria. For analyses, they were divided into two cohorts by treatment period: 2005–2014 cohort (*n* = 122 patients) corresponding to cases included in the original doctoral thesis study, and 2015–2024 cohort (*n* = 182 patients) representing the extended series. The study was conducted in accordance with the principles of the Declaration of Helsinki and approved by the Institutional Review Board of the Comité de Ética de la Investigación con Medicamentos Hospital General Universitario Gregorio Marañón (protocol code CMF2, approved on 19 October 2023). All participants provided informed consent prior to inclusion in the study, and data were handled with strict confidentiality in compliance with applicable data protection regulations.

### 2.2. Oncologic Treatment and Reconstructive Procedure

All patients underwent standard oncologic resection of their tumors, which in most cases involved partial mandibulectomy or maxillectomy and resection of adjacent tissues as necessary. Neck dissection was performed as indicated by tumor staging. The vast majority of oral cancers are squamous cell carcinomas, followed by less frequent histological types such as verrucous carcinoma, mucoepidermoid carcinoma, adenoid cystic carcinoma, osteosarcoma and others, but for simplicity, we refer to the cohort as oral cancer patients. The defect reconstruction was planned by a multidisciplinary team. In patients with segmental mandibulectomy or large maxillary defects, a microvascular free flap bone graft was typically used to restore jaw continuity and provide bone for implant placement [41]. In cases requiring bony reconstruction, the most frequently used microsurgical flaps were the free fibula flap and the iliac crest flap based on the deep circumflex iliac artery. In our series, about half of the patients in each cohort received a vascularized bone flap. For medium to large defects not involving osseous reconstruction, the radial forearm flap and the anterolateral thigh (ALT) flap were the most employed options. The remaining patients had smaller defects that were reconstructed with local flaps or allowed to heal secondarily, preserving enough native bone for later implant placement.

### 2.3. Primary Implant Placement

In select cases, dental implants were placed at the time of the tumor resection and reconstruction surgery. Primary implant placement was performed during oncologic surgery in selected cases with favorable anatomy—either due to the presence of sufficient residual native bone or when virtual planning enabled prosthetically guided implant positioning within the bone flap. This strategy was indicated in cases where the surgical margins were clear, the reconstructive site was well-vascularized, and the patient’s oncologic status and general health allowed for early implant placement as part of a comprehensive rehabilitation plan. No implant was immediately loaded with a prosthesis at the time of cancer surgery; even in primary cases, prosthetic rehabilitation would occur only after adequate healing (and after adjuvant therapy).

### 2.4. Secondary Implant Placement

Patients who did not receive implants during the initial surgery were considered for delayed implant placement once they had recovered from cancer treatment. Our institutional protocol was to wait a minimum of 6 months after completing radiotherapy (if applicable) before implant surgery, and more commonly around 12 months. For patients not receiving radiotherapy, a typical waiting time was 12–18 months after tumor resection to ensure there was no early recurrence. In many cases from 2005–2014, implants were intentionally delayed about 24 months post-surgery as an added precaution (given the highest risk period for locoregional recurrence is within the first 2 years). In the later cohort (2015–2024), with increasing clinician confidence, the waiting time was sometimes shortened (e.g., to 6–12 months post-op) if the patient was keen to proceed and had no evidence of disease. In most cases involving full-arch rehabilitation, multiple implant placements, severe trismus, risk of bronchial aspiration, or patient-related limitations for performing the procedure under local anesthesia and/or sedation, implant surgery was carried out under general anesthesia. If necessary, adjunctive procedures such as soft tissue grafts (vestibuloplasty or free gingival grafts) were performed concurrently to improve the peri-implant soft tissue conditions.

### 2.5. Implant Placement and Prosthetic Rehabilitation

Standard dental implant surgical protocols were followed, with modifications for the altered anatomy of these patients. We used titanium screw-type implants from Ticare Implants^®^, Valladolid, Spain. Ticare^®^ implants are fabricated from high-grade commercially pure titanium (Grade IV or Grade V ELI—Ti6Al4V-ELI), ensuring high mechanical strength and biocompatibility. The macrodesign of the implant features a conical body with a self-tapping thread pattern, intended to achieve high primary stability, even in areas of low bone density. One of the distinguishing characteristics of Ticare implants is the surface treatment, which is a thermochemical surface modification method. This treatment increases the surface roughness at the micro- and nanoscale, promoting early cell adhesion and osteoblastic activity, which accelerates osseointegration. Extensive in vitro studies have demonstrated that the implant–abutment connection system prevents bacterial infiltration, which is a critical factor in avoiding peri-implant inflammation and bone resorption. The tight conical connection and precision-machined interface play a fundamental role in achieving this hermetic seal. Implant dimensions were selected based on available bone, with lengths typically 8–15 mm and diameters 3.4–4.25 mm for anterior regions and up to 3.75–5.0 mm for posterior regions when bone width allowed. In irradiated patients, no routine hyperbaric oxygen therapy was employed; instead, prophylactic antibiotics and antiseptic rinses were used, and surgical technique was kept as atraumatic as possible (low-speed drilling with irrigation, minimal periosteal stripping). The number and distribution of implants per patient were planned in a prosthetically driven manner using pre-operative models or virtual planning. On average, 6–8 implants were placed to support a full-arch restoration in a resected jaw segment. In some extensive cases, up to 12–16 implants were placed across both jaws to support combined maxillary and mandibular prostheses.

**Figure 1:**
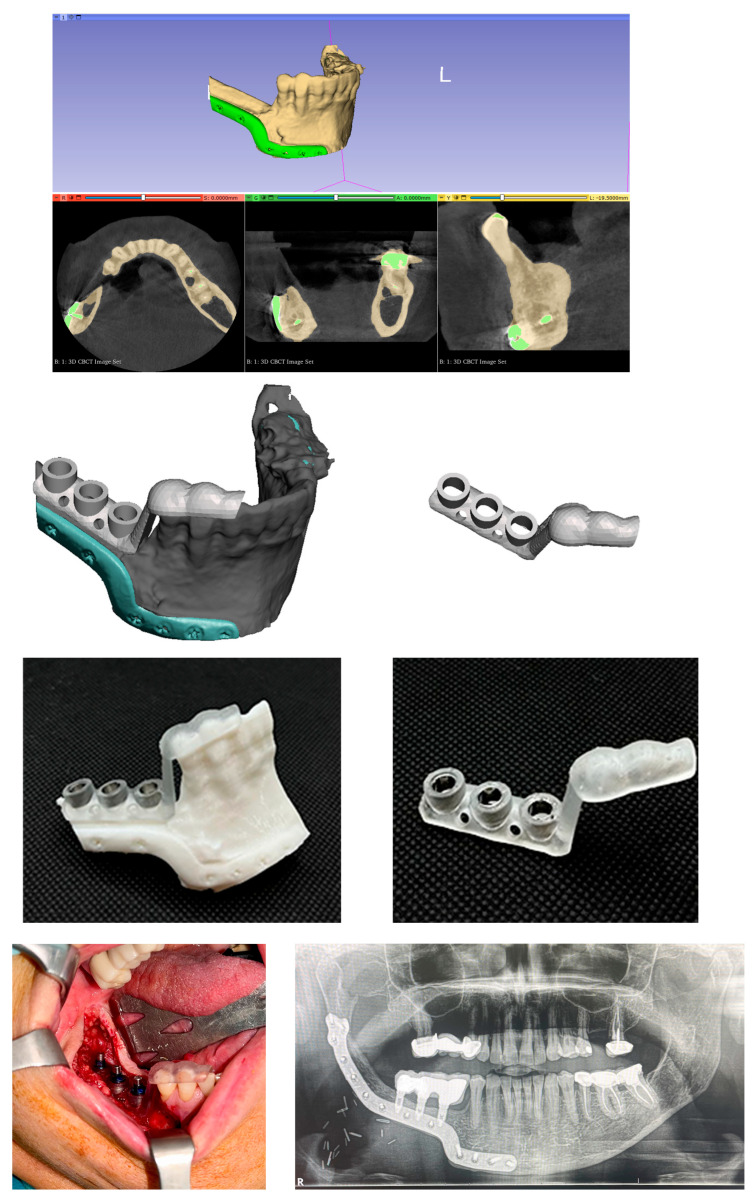
Example of an implant surgical guide manufactured through an academic point-of-care manufacturing workflow. The guide was designed for mandibular reconstruction using a fibula free flap and fabricated by stereolithography (SLA) technology with Biomed Clear V1 resin. Intraoperative view of the static guided implant surgical guide in position. Final panoramic radiograph (OPG) showing the completed case.

Since 2015, the department has integrated digital planning and 3D printing into the clinical workflow. This included the use of static guided implant surgery with custom surgical guides fabricated through a point-of-care (POC) manufacturing protocol, as well as the routine assessment of implant stability using the Implant Stability Quotient (ISQ) system. CT scans of patients were used to create 3D models of the jaws, and surgical guides were 3D-printed from biocompatible resin under POC manufacturing protocol [39,40] to assist in optimal implant positioning in complex cases (especially in reconstructed jaws). This point-of-care manufacturing approach improved the precision of implant placement and alignment with the intended prosthetic design (Figure 1).

After implant placement, a healing period was observed to allow osseointegration. In primary cases that also received radiotherapy, the healing period was inherently extended by the time needed for radiotherapy (6–7 weeks) and subsequent tissue recovery. In secondary cases, a typical healing time was 3–4 months for implants in native bone, and 4–6 months for implants in grafted bone (given potential reduced vascularity). Each implant was evaluated for osseointegration (via clinical stability and radiographic bone interface) before proceeding to prosthetic loading (second-stage surgery to place abutments, if two-stage implants were used). In some patients (especially those with primary placed implants), a one-stage approach was used with transmucosal healing abutments to avoid a second surgery.

### 2.6. Prosthetic Rehabilitation

Once osseointegration was confirmed—based on Implant Stability Quotient (ISQ) measurements—the prosthetic rehabilitation phase was initiated. The type of prosthesis (fixed vs. removable overdenture) was determined based on multiple factors: number and distribution of implants, jaw reconstructed contour, soft tissue conditions, patient’s oral opening and manual dexterity, and patient preference. Whenever feasible, a fixed implant-supported prosthesis was recommended, usually a screw-retained hybrid denture or bridge that replaces the teeth and missing tissues. In our practice, the majority of patients received fixed full-arch prostheses anchored by their implants [42]. In cases with limited implants or unfavorable positions, a removable overdenture retained by a bar or locator attachments was fabricated (this was more common in patients with very small mouth opening or those who could not maintain hygiene under a fixed bridge). Each prosthesis was delivered and adjusted for comfort and function. Patients and caregivers were instructed in meticulous oral hygiene, given the higher risk of peri-implantitis especially in irradiated fields.

We define successful rehabilitation as a patient receiving a functional implant-supported or implant-retained prosthesis. If a patient died or had disease recurrence before prosthetic treatment, or if for any reason the implants could not be used to fabricate a prosthesis, that patient was counted as not rehabilitated in our analysis (even if some implants were present). We also tracked the time intervals in treatment: from the date of cancer surgery to the placement of the first implant stage (in secondary cases) or to the placement of the prosthesis (in primary cases), and from prosthesis delivery to either the patient’s last follow-up or death. These times reflect the burden and speed of rehabilitation.

### 2.7. Data Collection and Outcome Measures

Patient records were reviewed for demographic data (age at surgery, sex), tumor characteristics (location, histology, TNM stage), treatment details (reconstruction type, radiotherapy dose and dates), and habits (smoking status). Implant-specific data included number of implants placed per patient, implant location (maxilla vs. mandible, anterior vs. posterior segment), timing of placement (primary vs. secondary), and whether implants were placed in native bone or a bone flap. Prosthetic data recorded included type of prosthesis (fixed vs. removable), date of prosthesis delivery, and any prosthetic complications.

The primary outcome measure was implant survival, defined as the implant remaining in situ with osseointegration at the last follow-up. Implant failure was defined as the loss or removal of an implant for any reason, including lack of osseointegration, infection, mechanical fracture, or other complications. Implants that were stable and in function (or stable at time of patient death) were considered surviving. We also applied the classical success criteria of Albrektsson et al. to characterize implant success: absence of mobility, no persistent peri-implant radiolucency, bone loss <0.2 mm annually after the first year of loading, and no peri-implant infection or pain [43]. An implant could be surviving but not “successful” if it had excessive bone loss or other issues; however, in this study, we primarily report survival rates, as detailed bone level data were not available for all patients.

The secondary outcomes included (a) patient-level rehabilitation success—whether the patient obtained an implant-supported prosthesis; (b) time to rehabilitation—measured as months from cancer surgery to prosthesis placement; (c) differences in implant survival based on subgroup factors (radiotherapy vs. not, jaw, region, timing); (d) occurrence of osteoradionecrosis (ORN) of the jaw related to implant placement—defined as non-healing bone necrosis in a previously irradiated site, occurring after implant surgery; and (e) prosthesis outcomes—particularly the proportion of patients receiving fixed prostheses versus overdentures, and any implant losses after prosthesis delivery.

Follow-up time was calculated from the date of implant placement to the date of last clinical follow-up or implant failure or patient death, as appropriate for different analyses. Patients were typically followed with regular exams every 3–6 months in the first 2 years and annually thereafter. Implant survival was censored as of 31 December 2024, which served as the study cutoff date. Patients without implant failure events were censored at the date of their last recorded clinical follow-up.

### 2.8. Statistical Analysis

Data were analyzed using IBM SPSS Statistics (v. 28) and Prism 9 (GraphPad). Continuous variables were summarized as mean ± standard deviation (SD) or median and range/interquartile range (IQR) as appropriate. Categorical variables were summarized as counts and percentages. Comparisons between the two cohorts (2006–2015 vs. 2016–2024) were made using the independent samples *t*-test for approximately normally distributed continuous variables (or Mann–Whitney U test for non-normal distributions such as time to prosthesis) and the chi-squared test (or Fisher’s exact test when counts were small) for categorical variables (e.g., proportion receiving radiotherapy or proportion rehabilitated). A two-sided *p* < 0.05 was considered statistically significant for differences between cohorts.

Implant survival rates over time were estimated using the Kaplan–Meier method. Survival curves were constructed for implants in each cohort, as well as for subgroups (e.g., irradiated vs. non-irradiated). The log-rank test was used to compare survival curves between groups. We also calculated cumulative 2-year and 5-year implant survival percentages with 95% confidence intervals. For patient-level analyses (such as “patient has all implants surviving”), additional Kaplan–Meier curves were generated (e.g., percentage of patients with no implant failures over time). However, the primary survival analysis treats each implant as an independent entity; we acknowledge that multiple implants are clustered within patients, but given the high survival, the dependency is minimal and was not adjusted for in Kaplan–Meier estimates.

All statistical tests were performed separately for the two cohorts and then compared. Additionally, multivariable analysis was not performed due to the limited number of failure events; instead, we relied on subgroup stratification for factors like radiotherapy and implant timing. The results are presented with relevant summary statistics and *p*-values, and tables/figures are provided to illustrate key findings.

## 3. Results

### 3.1. Patient Characteristics

A total of 304 patients (220 male, 84 female) were included, with 122 patients in the 2005–2014 cohort and 182 in the 2015–2024 cohort. The overall median age at tumor surgery was 60 years (range 24–82 years), with no significant age difference between the two cohorts (mean 60.5 vs. 61.0 years, *p* = 0.80). The vast majority of patients (80%) were male, reflecting the higher incidence of oral cancer in men with a history of tobacco and alcohol use. Indeed, about 89% of patients were active or former smokers, with a similar prevalence in both time periods (90.2% vs. 86.8%, *p* = 0.37). By diagnosis, >95% had squamous cell carcinoma of the oral cavity; a few patients had other oral malignancies, but these were evenly distributed, and all required similar rehabilitation.

Tumor treatment characteristics were also comparable between cohorts. Just over half of the patients in each group received adjuvant radiotherapy after surgery (52% in 2006–2015 vs. 50% in 2016–2024, *p* = 0.78). The typical radiotherapy regimen was 60–66 Gy in 30–33 fractions to the primary site and neck. The median radiation dose was 60 Gy in both cohorts (range 50–70 Gy, with some variation depending on margins and risk factors; *p* = 0.85). All irradiated patients had a minimum interval of 12 months between radiotherapy completion and implant surgery. Approximately 55–56% of patients underwent immediate free flap bony reconstruction of their jaw defect at the time of tumor resection, most commonly fibula flaps for mandibular defects. This rate did not differ significantly over time (55.7% vs. 54.9%, *p* = 0.90), indicating that the complexity of defects was similar in both cohorts. The remaining patients either had less extensive bony resection (not requiring a free flap) or, in a few cases, delayed secondary bone grafts (e.g., iliac crest graft) prior to implant placement.

Patients were followed for a median of 42 months (3.5 years) after implant placement (range 1 to 10+ years). By design, the early cohort had slightly longer follow-up (median 48 months) compared to the later cohort (median 36 months), since many later patients were more recently rehabilitated (*p* = 0.03 for difference in follow-up time). During the follow-up period, several patients died of their primary disease or other causes (the overall 5-year patient survival was ~65%, consistent with cancer statistics [43]). Importantly, 15 patients (12.3%) in the 2006–2015 group died or had recurrence before receiving any dental prosthesis, whereas only eight patients (4.4%) in the 2016–2024 group failed to reach prosthetic rehabilitation due to death or disease progression. This difference in rehabilitation rate was statistically significant (*p* ≈ 0.01), suggesting improved oncologic or supportive care in the latter period such that more patients survived to complete dental rehabilitation.

Table 1 summarizes the baseline characteristics of the two cohorts. There were no significant differences in age, sex distribution, smoking status, or radiotherapy exposure between the cohorts, indicating they were suitable for comparison of outcomes. The slight difference in follow-up duration reflects the study design but was accounted for in survival analyses via censoring.

### 3.2. Implant Placement and Rehabilitation Parameters

In total, 2341 Ticare Implants^®^ were placed in the 304 patients. The 2006–2015 cohort received 941 implants, and the 2016–2024 cohort received 1400 implants. The number of implants per patient ranged from two (in a few limited maxillary cases) to 16 (in a patient who had both jaws reconstructed and fully implanted). The mean ± SD number of implants per patient was 7.7 ± 3.4 in the first cohort and 7.5 ± 3.1 in the second cohort, which was not a significant difference (*p* = 0.60). Thus, the extent of implant rehabilitation (in terms of implant count) remained similar over time, reflecting comparable defect sizes and prosthetic needs.

One of the most notable differences between the two cohorts was the timing of implant placement. In the 2006–2015 group, only 50 patients (41%) received any implants during the primary cancer surgery, whereas in the 2016–2024 group, 130 patients (71%) had immediate implants placed at the time of tumor resection (primarily in conjunction with fibula flap reconstructions). This increase in primary implant utilization from ~40% to ~70% of cases is highly significant (χ^2^, *p* < 0.001). It indicates a shift in practice after 2015 towards favoring immediate implant placement when feasible. By contrast, the number of patients undergoing purely secondary (delayed) implant placement decreased in proportion. Those who did receive secondary implants had a shorter waiting interval on average in the later cohort (median ~12–15 months post-surgery) than the earlier cohort (often ~24 months post-surgery), although this specific interval was not uniformly recorded for statistical comparison.

Because of the greater use of immediate implants, the overall time from cancer surgery to prosthetic rehabilitation was markedly reduced in the 2016–2024 cohort. For each patient, we measured the interval from the date of tumor resection to the date of prosthesis placement. In the first cohort, this time ranged widely depending on treatment course; the median time to rehabilitation was 24 months (2 years), with an interquartile range of 18–36 months. In the second cohort, the median time to prosthesis was 15 months, IQR 9–24 months. This represents a difference of approximately 9 months (median) in favor of the later cohort. The reduction is statistically significant (Mann–Whitney *U*, *p* < 0.001). Figure 1 illustrates the cumulative proportion of patients rehabilitated over time for the two cohorts. By 2 years after surgery, about 48% of patients in the 2006–2015 cohort had received their implant prostheses, compared to 72% of the 2016–2024 cohort. By 3 years post-op, 90% vs. 98% were rehabilitated in the two groups, respectively. This confirms that the second cohort achieved functional dental restoration significantly faster on average than the first cohort.

The proportion of patients who were successfully rehabilitated with an implant-supported prosthesis improved between cohorts. In the first cohort, 107 out of 122 patients (87.7%) ultimately received an implant-based dental prosthesis (fixed or removable), while 15 patients (12.3%) never did (due to early death, surgical complications, or tumor recurrence preventing rehabilitation). In the second cohort, 174 out of 182 patients (95.6%) completed implant prosthetic rehabilitation, and only eight patients (4.4%) did not. This difference—essentially the “rehabilitation success rate”—was statistically significant (*p* = 0.01). The improvement implies that in recent years, a higher fraction of patients who embark on the implant process make it to the finish line with a functional prosthesis, likely owing to better patient selection and perhaps improved cancer survival in the short term.

Table 2 summarizes key treatment and outcome metrics for the two cohorts. It highlights the significant increase in primary implant placement and the reduction in time to prosthesis, as well as differences in early failure rates and rehabilitation success.

As seen in Table 2, the early implant failure rate (implants that never achieved osseointegration, failing prior to prosthetic loading) was significantly higher in the 2005–2014 cohort. Out of 941 implants placed in that cohort, 37 failed during the healing phase, a rate of 3.9%. In contrast, in the 2015–2024 cohort only 28 of 1400 implants failed early, a rate of 2.0% (*p* = 0.03 for difference). Thus, the later cohort had roughly half the incidence of early implant loss. This likely reflects improvements in surgical technique and patient management (e.g., more precise placement reducing micromotion, better primary stability, and improved systemic care) over time. It is noteworthy that about two-thirds of these early failures (25 of 37 in the first cohort) occurred in irradiated patients, suggesting radiation compromised early healing in some cases, although overall irradiated vs. non-irradiated survival still showed no long-term difference (owing to low numbers and successful later management of many radiated cases).

By study cutoff, many implants had long-term follow-up available. The cumulative 5-year implant survival (all causes) was 95% in the first cohort (95% confidence interval ≈ 92–97%) and 97% in the second cohort (CI ≈ 95–99%). This small improvement was not statistically significant (log-rank *p* = 0.30), but it aligns with the trend of fewer failures in the second period. At 2 years post-placement, implant survival was 97% vs. 99% in the first vs. second cohort (difference < 2%). The Kaplan–Meier curves for implant survival (Figure 1) show that most implant losses occurred early (within 6–12 months) in both cohorts; after the first year, the survival curves plateaued, indicating very few late failures in either group. Only a handful of implants were lost after prosthetic loading in each cohort (detailed below), hence the 5-year survival closely mirrors the early integration success.

### 3.3. Implant Survival by Subgroup Factors

We further analyzed implant survival with respect to radiotherapy, jaw location, and implant timing. In agreement with our earlier study, there was no statistically significant difference in implant survival between irradiated and non-irradiated patients in our data. In the 2005–2014 cohort, 55 patients had received radiotherapy (with 512 implants in irradiated bone), and 52 patients had not (333 implants in non-irradiated bone) [9]. The 5-year implant survival was ~94% in irradiated vs. ~96% in non-irradiated cases (not significant, *p* ≈ 0.5). Similarly, in the 2015–2024 cohort, irradiated cases (91 patients, ~750 implants) had ~97% 5-year survival vs. ~98% in non-irradiated cases (no significant difference). Our findings reinforce that, with proper precautions, radiotherapy does not preclude high implant success rates, echoing reports in the literature that careful patient selection and management can yield similar outcomes in irradiated patients [19,44]. It should be noted, however, that irradiated bone was associated with a higher early failure count (as mentioned, 25 of 37 early failures in the first cohort were in irradiated bone), even though those that survived the initial period tended to remain stable long-term.

**Figure 2:**
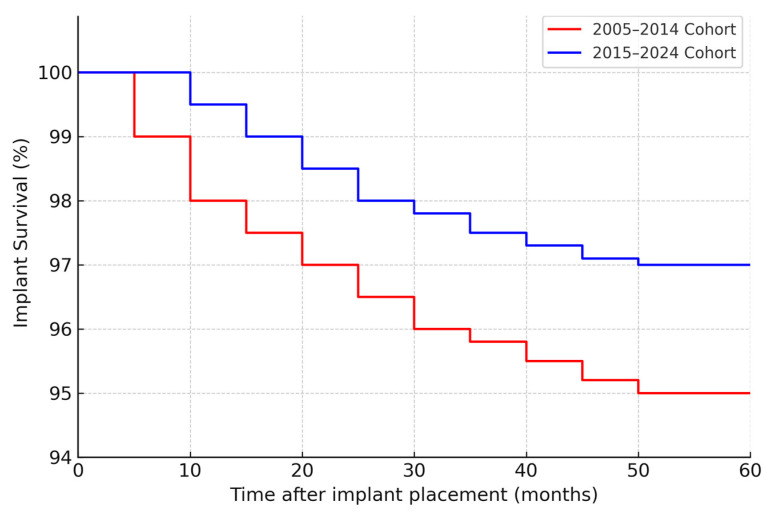
Kaplan–Meier curves showing the implant survival over time in the two cohorts (2005–2014 in red, 2015–2024 in blue). The *Y*-axis represents the percentage of implants remaining successfully integrated (not lost) as a function of time since implant placement. Both cohorts exhibit high survival, but the 2015–2024 cohort shows a slightly higher survival curve, especially in the early post-implant period. By 60 months (5 years), survival was ~95% in the 2005–2014 group vs. ~97% in the 2015–2024 group (log-rank *p* = 0.30, n.s.). The initial steeper drop in the red curve reflects a higher rate of early implant failures in the 2005–2014 cohort.

Regarding anatomical location, in the 2005–2014 cohort, we observed significantly different failure patterns (Figure 2). Implants placed in the mandible had a higher failure rate than those in the maxilla (overall 5-year survival ~93% vs. 98%, *p* ~0.01), and implants in posterior segments (molars/premolar regions) had more failures than those in anterior segments (incisor/canine regions) (survival ~92% vs. 99%, *p* ~0.01). These differences were especially pronounced for irradiated patients: for example, in irradiated cases, posterior mandibular implants were the most failure-prone. Most of these failures occurred early, as reflected in the significantly lower primary integration success in posterior mandible positions. In the 2015–2024 cohort, by contrast, we did not find a statistically significant difference between mandibular and maxillary implant survival, nor between anterior vs. posterior (log-rank *p* > 0.1 for both comparisons). This suggests that improved surgical planning and implant distribution in later years mitigated the site-specific risks. For instance, the use of computer-guided placement might have helped avoid certain unfavorable positions or allowed placing additional implants in high-risk posterior areas to ensure redundancy. Moreover, many mandibular cases in the later cohort had fibula flaps with primary implants—a combination that yielded very high success (nearly 99% 1-year survival in the literature [33] and similar in our experience).

When considering primary vs. secondary implants, we found a slight difference in favor of primary placement, but it was not statistically significant in terms of implant survival. Pooling both cohorts, primary-placed implants had a 5-year survival of ~98% versus ~95% for secondary-placed implants (*p* = 0.20, n.s.). In the first cohort, primary implants showed a trend toward fewer failures (e.g., 2-year success ~99% for primary vs. 96% for secondary), but the sample was not large enough for significance. The second cohort had relatively few secondary implants, and their survival (~96% 5-year) was on par with the primary group (~97%). These data indicate that immediate implant placement did not negatively impact implant success; if anything, outcomes were equally good or better compared to delayed placement. This aligns with recent studies advocating primary implantation during free flap reconstruction, which found no survival disadvantage and potentially earlier functional benefits [45].

### 3.4. Prosthetic Outcomes and Complications

Out of the patients who were rehabilitated, the majority received fixed implant-supported prostheses. In the 2005–2014 cohort, 85 of 107 rehabilitated patients (79%) were provided with fixed screw-retained prosthetic restorations (either full-arch bridges or large partial bridges). The remaining 22 patients (21%) received removable overdentures retained by implants (generally on bars or ball attachments). In the 2015–2024 cohort, a significantly higher proportion received fixed prostheses: 160 of 174 patients (92%) had fixed implant-supported bridges, while only 14 patients (8%) were restored with overdentures. The increase in usage of fixed prostheses (79% → 92%) is significant (*p* = 0.002). This reflects a trend towards more optimal rehabilitation, as fixed prostheses are usually preferred for better function and patient comfort when conditions allow. The reasons some patients still required overdentures included extremely limited mouth opening (trismus/fibrosis post-radiation, making hygiene under a fixed bridge impractical), insufficient number or distribution of implants (in a few cases of partial rehabilitation), or patient preference in rare instances. Notably, some patients initially received overdentures as an interim solution but later transitioned to fixed prostheses as additional implants were placed or conditions improved; for the purpose of analysis, we counted the final long-term restoration.

The functional outcomes were generally excellent. Patients with fixed prostheses were able to chew a broad diet and had significant improvements in speech intelligibility and social confidence, as documented in follow-up notes (subjective quality-of-life outcomes will be reported separately). Those with overdentures also benefited greatly compared to being edentulous, though some had minor complaints of prosthesis movement or need for periodic relines.

Implant losses after prosthetic loading were very rare in our series. In the 2005–2014 cohort, only two implants (out of 845 that had been loaded in prostheses) were lost during the post-loading follow-up period, an incidence of 0.24%. In the 2015–2024 cohort, one implant (out of ~1330 loaded) was lost after loading (~0.08%). These late failures were likely due to peri-implant infection (peri-implantitis) or occlusal overload. All occurred in patients who still had sufficient other implants to retain their prosthesis, and the situations were managed by either replacing the lost implant or modifying the prosthesis to cantilever off the remaining implants. No catastrophic prosthetic failures occurred (no patient went back to being edentulous once rehabilitated).

The occurrence of osteoradionecrosis (ORN) related to dental implants was exceedingly low. We observed only one case of ORN in the entire study: a patient in the first cohort (irradiated mandible) developed a localized mandibular ORN around an implant site. This case was managed successfully with conservative measures (antibiotics, local debridement, Pentoxifylline and Tocopherol medication), and the patient’s implants were ultimately removed as part of resolving the necrosis. In the second cohort, no ORN cases were encountered associated with the implant surgeries. The overall ORN incidence in irradiated patients who received implants was ~1 out of 154 irradiated patients (~0.65%). This is well below the 3–5% ORN incidence reported in some earlier studies for post-implant ORN [45,46]. Our low ORN rate likely reflects careful adherence to surgical protocols (e.g., avoiding incisions in previously irradiated tissue when possible, perioperative antibiotics) and perhaps the selection of healthier irradiated patients for implants. Additionally, all irradiated patients had stable mucosal coverage before implant placement and did not receive implants in heavily dose-compromised bone without hyperbaric oxygen or other preparation. The finding is encouraging, as ORN is a serious complication; fortunately, in our series, it was essentially anecdotal. 

Peri-implant mucositis and peri-implantitis represent relevant long-term risks, particularly in patients who have received radiotherapy or exhibit compromised salivary function. Although late implant loss due to peri-implantitis was infrequent in our cohort (<0.2%), structured follow-up protocols were critical. All patients were enrolled in a dedicated implant maintenance program, which included clinical evaluation, hygiene reinforcement, and periodic radiographic monitoring. ISQ values were recorded not only at placement but during follow-up to detect early signs of stability loss. Topical chlorhexidine, custom hygiene devices, and frequent debridement were employed for high-risk patients. Future studies should focus on integrating objective biomarkers (e.g., peri-implant crevicular fluid analysis) and AI-assisted imaging tools to improve early detection of peri-implant disease in oncologic populations. In fibular flap reconstructions, management of the peri-implant soft tissue interface plays a crucial role in achieving long-term stability and reducing peri-implant complications. Given the frequent mismatch between flap thickness and ideal mucosal contours, surgical soft tissue refinement was routinely performed. Techniques such as vestibuloplasty, free gingival grafting, and soft tissue debulking were employed when necessary to establish a stable, keratinized mucosal seal around the abutments. These procedures were typically planned during the second-stage surgery or early in the prosthetic phase, ensuring optimal peri-implant health and facilitating long-term maintenance.

Finally, virtually all implants that achieved osseointegration were ultimately used to support prostheses. In the first cohort, thanks to prosthetically guided planning, only about 1.7% of integrated implants ended up not being connected to a prosthesis (for example, an implant that integrated but was later judged to be in a suboptimal position or angulation such that it could not be included in the bridge framework). This was achieved by careful secondary placement strategies. In the second cohort, with digital planning and improved accuracy, this figure was even lower—only 0.5% of integrated implants were not utilized. Essentially every implant placed was part of the final functional reconstruction, maximally leveraging the surgical effort.

In summary, the results demonstrate that both cohorts achieved high success in terms of implant integration and prosthetic function, with the later cohort showing several improvements: a higher rehabilitation rate, faster timeline to teeth, slightly fewer implant failures, and more patients receiving the ideal fixed restorations (Figure 3, Figure 4, Figure 5, Figure 6, Figure 7, Figure 8, Figure 9, Figure 10, Figure 11, Figure 12, Figure 13 and Figure 14).

## 4. Discussion

This study presents one of the largest single-center experiences to date involving dental implant rehabilitation in oral cancer patients, encompassing 304 patients over a 20-year period. The outcomes strongly support the feasibility of implant-supported prosthetic restoration in this challenging patient population. Overall, our implant survival rate (~95–97% at 5 years) is comparable to outcomes in healthy patients reported in the general implant literature [9,47,48]. For context, standard implant success in non-oncologic individuals is often cited as around 95% at 5–10 years, and our data suggest that—with proper protocols—oral cancer survivors can achieve similarly excellent implant longevity.

The placement of dental implants in oral cancer patients who have undergone radiotherapy is increasingly recognized as a viable rehabilitation option, though it presents unique challenges and considerations [49]. Multiple studies demonstrate that implant-based oral rehabilitation is feasible in irradiated patients, with overall success rates ranging from 86% to 97%, depending on radiation exposure and patient-specific factors [50,51]. Although earlier radiotherapy was historically seen as a contraindication, recent evidence supports implant placement in irradiated bone when appropriate precautions and follow-up are ensured [52]. Critical factors influencing implant success include the radiation dose, with higher failure rates observed when doses exceed 50 Gy [53], and implant location, where mandibular implants generally show higher survival than maxillary implants [38]. Delayed loading protocols, where implants are left unloaded for at least 6 months, are associated with significantly better osseointegration outcomes [54].

Primary implant placement during oncologic surgery has also been shown to provide favorable results, reducing the need for additional surgeries and expediting rehabilitation [55]. However, long-term risks such as peri-implantitis, impaired healing due to soft tissue damage, and osteoradionecrosis must be considered [55]. Moreover, systematic reviews suggest that despite high medium-term survival rates, further high-quality research, including randomized clinical trials, is still needed to strengthen evidence and refine clinical guidelines [56].

One of the key findings is that adjuvant radiotherapy did not significantly diminish implant success in our series. The implants in irradiated bone had only a slightly lower cumulative survival (around 1–3% difference) than those in non-irradiated bone, and this difference was not statistically significant. This aligns with several recent studies that have also reported no major impact of radiation on implant survival when confounding factors are controlled [57]. For example, a meta-analysis focusing on 2007–2013 data found no significant difference between implants in irradiated vs. native bone [58], which mirrors our findings. On the other hand, older reports in the literature and some meta-analyses have shown a clear effect of radiation, with reported survival rates of ~84–92% in irradiated cases vs. ~95% in non-irradiated [19,31,59,60,61]. Chrcanovic et al. (2016) noted a risk ratio indicating significantly higher failure in irradiated patients [12].

A more recent systematic review (2021) calculated a pooled implant survival rate of 91.9% in irradiated head-neck cancer patients, which is somewhat lower than what we observed in our cohort (roughly 95% at 5 years for irradiated) [10]. The differences may be due to the stringent care and selection in our practice: we excluded patients with very high-dose implant-bed radiation or very poor tissue quality, we ensured a healing period before implantation, and we maintained aggressive oral hygiene and follow-up. Additionally, the timing of implantation relative to radiotherapy might influence outcomes. In our series, a number of irradiated patients had primary implants placed before radiotherapy (i.e., implants placed at surgery, then the area irradiated). Some reports in the literature suggest implants placed prior to radiotherapy may fare better than implants placed into already irradiated bone, because the implant can integrate before radiation-induced fibrosis sets in. Though we did not specifically stratify by this sequence, it could partly explain our high success in irradiated cases.

Another notable observation was the extremely low incidence of osteoradionecrosis (ORN) (0.65%). This is an important safety outcome. Implants in irradiated jawbone have been historically feared as potential triggers for ORN, with earlier reports advising caution. Our data, along with other modern studies, indicate that the risk is very low, especially when implants are placed in a controlled, atraumatic manner with adequate healing time [62,63]. A meta-analysis found an ORN incidence of ~3% among irradiated implant patients, and another review noted most ORN cases are limited and manageable [10,12,64,65,66]. The single ORN case in our cohort resolved with conservative treatment, and no implants had to be removed due to ORN except in that case. This reassures that concerns about ORN should not be a contraindication to implant rehabilitation, provided the standard precautions are taken (such as good periodontal health, avoidance of implant placement in extremely high-dose regions like within previous brachytherapy seeds, etc.). Our practice did not use hyperbaric oxygen therapy (HBOT) prophylactically, consistent with mixed evidence on HBOT efficacy for dental extractions/implants in irradiated patients. Instead, we relied on surgical skill and Pentoxifylline/Vitamin E in cases of early signs of ORN, which likely contributed to prevention and healing [67,68,69,70].

The timing of implant placement relative to cancer treatment is an area where our study provides valuable insight. We saw a clear institutional shift toward primary implant placement after around 2015, which correlated with improved patient outcomes. Primary implant placement during the tumor resection and reconstruction offers the advantage of combining surgeries (reducing additional anesthetic events) and placing implants in an ideal trajectory under direct vision or guided by pre-planning. In cases of fibula flaps, it allows for a “one-step” reconstruction where the patient wakes up with implants already in the new jaw, dramatically shortening the path to final teeth. Our results showed no penalty in implant survival for primary placement—in fact, primary implants tended to have equal or slightly better success than secondary implants (albeit not significantly different). This finding echoes other reports: for instance, a recent meta-analysis [33] focusing on fibula flap cases found 1-year survival ~98% for immediate implants vs. 97% for delayed, with no significant difference [33]. Other authors similarly found that primary implants can integrate well and allow faster rehabilitation [28,71]. The major benefit we documented was the reduced rehabilitation time: by increasing primary implants, we cut the median time to prosthesis from 2 years down to just over 1 year. This is a meaningful improvement for patients, who regain function and normalcy much sooner. It also potentially reduces the window during which tumor recurrence might occur before the patient receives teeth (a situation that unfortunately affected some in the early cohort who died within those 2 years waiting).

It is important to note that primary placement is not universally applicable. In our early cohort, only 41% received primary implants largely because many surgeons were cautious—at that time, it was a relatively novel approach. By the later years, experience and emerging evidence gave surgeons more confidence to perform it in ~70% of cases. Still, about 30% of patients in recent years did not receive primary implants; reasons include very advanced disease where adding implant time was not prudent, patient’s general condition (some could barely tolerate the oncologic surgery itself), or logistic issues (lack of pre-op planning time for an immediate implant guide if a patient came urgently). For those cases, secondary implants remain effective, just that patients must endure a longer waiting period edentulous or with an obturator denture. Our data showed that secondary implants, when placed after ~1–2 years, also had excellent integration (~95% at 5 years), confirming that even delayed rehabilitation is worthwhile if immediate is not possible.

A significant difference we observed was in the influence of jaw location on implant success and how this changed with improved techniques. Initially, we found the mandible (especially posterior mandible) had higher failure rates than maxilla/anterior. This contrasts with some general implant studies where maxillary implants (especially in anterior maxilla with poorer bone quality) can have slightly lower success. In head-neck cancer patients, the scenario is different: the posterior mandible often corresponds to the area of the mandibular body or angle that might have received a fibula graft or been heavily irradiated, and is subject to high masticatory forces. In our early cohort, indeed, posterior mandible implants (particularly in irradiated bone) were the most vulnerable (*p* = 0.009–0.019). We responded to this finding in later cases by strategizing differently: for example, placing an increased number of implants in the posterior mandible to distribute forces (if anatomy allowed), or using longer implants engaging basal bone. Additionally, the use of angled multi-unit abutments and careful prosthetic design minimized cantilever forces on distal implants. Consequently, in the 2016–2024 group, site-specific differences disappeared statistically. This improvement highlights the value of learning curve and adaptive technique—a phenomenon likely present in our study since the same surgical team refined their approach over 20 years. It also implies that what used to be considered a “high-risk zone” can become effectively managed with current methods.

The prosthetic outcomes in our series are particularly encouraging. Over 90% of patients in recent years were able to receive a fixed implant prosthesis. Having a fixed bridge that is permanently anchored (yet retrievable with screws for maintenance) is generally superior for function compared to a removable denture, especially for younger or active patients. The trend from 79% to 92% fixed rate suggests that improvements in implant placement (accuracy and number) allowed more patients to have a fixed solution when previously they might have had to settle for an overdenture. This also reflects possibly better flap reconstruction that resulted in more favorable ridge anatomy for fixed prosthetics. Our fixed prostheses were mostly hybrid dentures (acrylic teeth on a metal framework) or porcelain-fused-to-metal bridges, depending on the case. Overdentures were reserved for cases where cleansability was a major issue or insufficient implants could be placed. In the early cohort, certain irradiated patients were given overdentures out of caution to allow easy removal and inspection of tissues (fear of ORN under fixed bridges). With growing trust in implant stability, we now rarely do that; if implants are solid, we commit to fixed and just follow the patient closely.

The low rate of post-loading implant loss (late failures) in both cohorts indicates that once implants survive to support a prosthesis, they tend to remain healthy. This speaks to the success of maintenance protocols. Patients were kept on regular follow-up and instructed intensely in hygiene measures. Many irradiated patients have xerostomia and are at risk of peri-implant infection; our team worked with dental hygienists to schedule frequent cleanings and topical fluoride to prevent caries in remaining teeth and mucositis around implants. As a result, late peri-implantitis leading to failure was extremely uncommon (just a few implants). This outcome is better than some general population studies where peri-implantitis rates can be higher; possibly our shorter average follow-up (only a subset have 10-year follow-up) partly accounts for it, but also, these patients, having gone through so much, are often very motivated to care for their implants.

### 4.1. Limitations

This study is retrospective, so it inherits limitations of that design. We did not randomize patients to primary vs. secondary implants; thus, some differences could be due to selection bias (surgeons likely chose primary implants for healthier or less advanced cases, which themselves might have better outcomes independent of timing). However, the large sample and consistent protocols mitigate some biases. Another limitation is the difference in follow-up time—later cohort patients naturally have had shorter follow-up so far. We used Kaplan–Meier analysis to handle censoring, but long-term comparisons (e.g., 10-year outcomes) are currently not equal. It is possible that as more time passes, the later cohort might experience some late failures that bring their survival curve closer to that of the earlier cohort. That said, given the pattern of implant failure (mostly early), we suspect the majority of risk differences have manifested. We also did not perform a multivariate regression due to relatively few failure events (only ~80 total implant failures out of 2341 Ticare Implants^®^, which is an event rate <4%). This makes it hard to statistically tease out independent predictors with high confidence. Instead, we relied on subgroup comparisons. For instance, radiotherapy and jaw site are somewhat confounded (more radiated mandibles in our sample), but by analyzing separately and observing improvements in one group vs. not in another, we can infer where improvements had effect.

Additionally, while we report high-level prosthetic outcomes (fixed vs. removable, success vs. not), we did not quantify functional scores or patient-reported outcomes here. In future analyses, we plan to present detailed quality-of-life questionnaire results, which are crucial for demonstrating the true benefit to patients. Nonetheless, even without formal questionnaires, the clinical endpoints of obtaining a functional set of teeth and being free of major complications are valuable measures that correlate with patient satisfaction.

### 4.2. Clinical Implications

The findings from our study reinforce that implant-based rehabilitation should be considered the standard of care for suitable oral cancer patients following ablative surgery. Historically, some centers were hesitant to pursue implants in these patients due to fear of complications or the assumption that outcomes would be poor. Our data, along with growing evidence, dispel these notions—showing that success rates are high and complications minimal, even in the context of radiation and free flaps [72,73,74]. Key practices that contributed to success in our program include the following:

- Multidisciplinary planning: Every case was discussed with oncologic surgeons, prosthodontists, and often radiologists. This ensured implant positions were planned in relation to the final prosthetic needs (prosthetic-driven approach). For example, we often inserted implants in the fibula flap segment in ideal alignment for a bridge, even if that meant extra surgical time [75].
- Timing personalization: We increasingly used primary placement for favorable cases, expediting rehab. But we still employed secondary placement for high-risk situations, thereby not forcing implants in all cases but tailoring to patient condition.
- Technical precision: Using 3D guides from 2015 onward improved implant positioning and likely reduced failures. The introduction of point-of-care 3D printing at our hospital allowed rapid production of patient-specific guides and models, which was particularly helpful for unusual anatomies. This technology likely also contributed to reducing the small percentage of mis-positioned implants (from 1.7% to 0.5% unused implants).
- Adherence to oncologic safety: We maintained the principle that cancer treatment comes first. Implants were only placed when they did not compromise oncologic resection margins or delay necessary therapies. In secondary cases, we ensured the patient had no evidence of disease (NED) at the time of implant placement (usually via recent scans). This is why only a small fraction (3%) of patients had recurrence after starting implants, and fortunately, none of those recurrences were attributed to the implant process. By waiting 1–2 years or performing adequate surveillance, we avoided “wasted” efforts in most instances.
- Patient training and follow-up: Because these patients often have special needs (e.g., trismus, altered anatomy), we provided custom tools like modified toothbrushes and water-irrigation devices for implant hygiene, and scheduled maintenance visits more frequently than for a typical implant patient. This proactive approach likely prevented late peri-implant problems.

In comparing our two cohorts, we essentially witnessed the evolution of care over two decades. The latter cohort enjoyed the benefits of incremental improvements made possible by learning from the earlier cohort’s outcomes. This manifested in concrete metrics: fewer implants failing, quicker rehabilitation, more fixed teeth, etc. It is gratifying to see that hypothesis-driven changes (like increasing primary implants and implementing digital planning) indeed translated into better outcomes.

Our results also serve to benchmark what is achievable in a high-volume center. Implant survival of around 95–97% at 5 years in oral oncologic patients is among the highest reported. For instance, a large systematic review reported an overall implant survival in oral cancer patients of ~89.9%, with a wide range across studies [5,10,12,13,30,33,36,37]. The authors noted significant heterogeneity and improvements in recent studies. The fact that our later cohort topped ~97% at 5 years suggests that the gap between cancer patients and healthy patients can be nearly closed. We attribute this to rigorous patient selection (e.g., excluding heavy ongoing smokers from implants until they quit, as smoking is another independent risk factor) [24] and improved techniques. Many of our patients were indeed heavy smokers (almost 90%), which usually predicts worse implant outcomes, yet our survival remained high. Perhaps those who continued heavy smoking did not live long enough to affect implant outcomes drastically, or they had lower implant counts (some data suggest smokers have slightly reduced implant survival).

Interestingly, our study also highlights the impact of surgeon experience. All implants in both cohorts were placed by a consistent surgical team at the same institution, removing operator variability. Thus, improvements can be largely attributed to increased experience and adoption of new technology by that same team. Other institutions starting such a program may initially see lower success but can achieve these outcomes as experience grows. It underscores the importance of specialized training when dealing with irradiated and reconstructed patients, as they are more complex than routine implant cases.

In terms of limitations, one could point out that our study is single-center, and results may not generalize to centers with less resources or different patient demographics. For instance, all our free flap surgeries were performed by seasoned microvascular surgeons, enabling optimal bone placement for implants; a center that performs fewer flap procedures might not place bone in as ideal a position, making it harder for implants to succeed. Additionally, our patients were mostly European; in other populations (with different co-morbidities or tumor biology), results could vary.

Looking forward, one area to explore is the use of Zygomatic implants or other novel implants for cases with extensive maxillary defects or in cases of ORN to rehabilitate when conventional implants might not be viable. Another aspect is the integration of implant loading protocols: in healthy patients, immediate loading of implants is popular, but in oncologic patients, we have been cautious. Perhaps in the future, with improvements in implant surface technology and imaging, some patients could receive their fixed provisional teeth much sooner (even within days of surgery in selected cases), which would further improve quality of life. Pilot cases of “teeth-in-a-day” or ‘’jaw-in-a-day’’ even for cancer patients have been described, but long-term data are needed [28].

Finally, our findings strongly support the idea that multidisciplinary collaboration is the critical determinant of successful outcomes. The statement that “implants have represented a revolution for oncologic patients”, originally cited in a Spanish doctoral thesis [9], is substantiated by our data. Patients who would have previously been left with a marginal mandibular denture—or no dentition at all—can now receive fixed prostheses anchored in fibula bone grafts, thereby restoring near-normal oral function. This advancement not only enhances diet and nutritional status but also improves psychosocial well-being, enabling survivors to speak, smile, and engage socially with greater confidence after overcoming cancer.

In conclusion, our expanded analysis confirms and extends the original findings: implant-supported oral rehabilitation is not only possible but highly successful in oral cancer patients, even those who undergo radiotherapy. The integration of immediate implant placement during ablative surgery, digital surgical planning, and guided protocols has led to improved efficiency and higher rates of fixed prosthetic delivery. The observed 5-year survival rate exceeding 95%, with over 90% of patients achieving functional rehabilitation, supports this approach as a standard of care. These results are particularly significant in the context of oncologic patients, who historically faced limited reconstructive options. Our findings underscore the need for early multidisciplinary planning and support the incorporation of implant protocols in the primary oncologic workflow. Continued advances in point-of-care manufacturing, implant surface technology, and patient-specific prosthetic planning are expected to further enhance outcomes and reduce rehabilitation time. This study provides a robust benchmark for institutions seeking to optimize functional recovery and quality of life in head and neck cancer survivors.

## 5. Conclusions

Within the limitations of this 20-year retrospective study, we draw the following conclusions:

Implant-supported oral rehabilitation is a predictable and durable solution for head and neck cancer patients. In our cohort of 304 patients, 5-year implant survival exceeded 95%, and over 90% achieved successful functional restoration—outcomes comparable to those in non-oncologic populations.

Adjuvant radiotherapy did not significantly compromise implant survival. With appropriate patient selection and surgical planning, irradiated bone supported implants with similar long-term outcomes (~95%) and a very low incidence of osteoradionecrosis (<1%).

Immediate (primary) implant placement during tumor resection significantly shortened the time to prosthetic rehabilitation without compromising implant survival, supporting its use when oncologically and technically feasible.

Implants placed in vascularized bone grafts (e.g., fibula, iliac crest) performed as well as those in native bone, reinforcing their reliability for osseointegration and long-term function.

The majority of patients received fixed implant-supported prostheses, which restored near-normal oral function and quality of life. Removable prostheses were reserved for selected cases with anatomical or functional limitations.

Over the two decades, technical and procedural advances—such as guided surgery, improved implant planning, and multidisciplinary coordination—led to a reduction in early failures, faster rehabilitation, and increased delivery of optimal fixed prosthetic solutions.

In summary, implant-supported rehabilitation is a safe and effective standard of care following oral cancer surgery. Continued innovation in immediate placement, digital planning, and prosthetic protocols is expected to further enhance outcomes. We advocate for early integration of implant planning into oncologic treatment pathways to ensure that survivors regain function, esthetics, and quality of life.

## Figures and Tables

**Figure 3:**
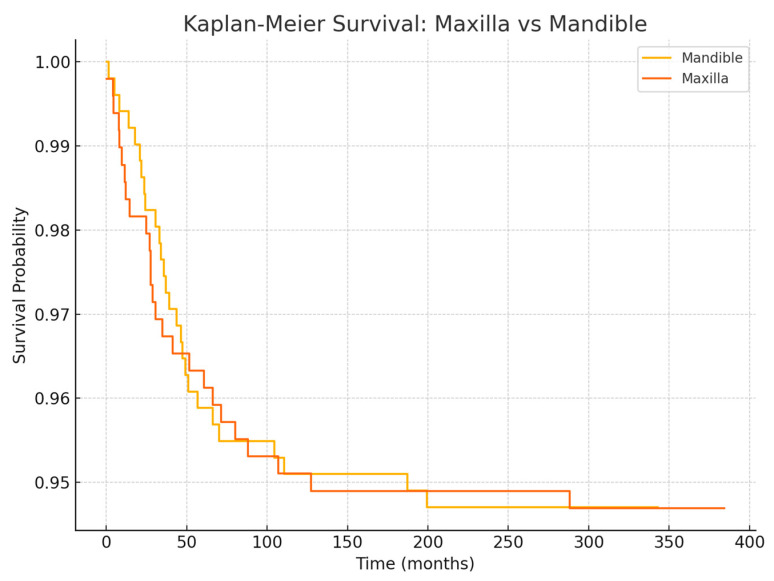
Kaplan–Meier survival curves comparing dental implant survival between maxillary and mandibular bone sites. Differences between groups were assessed using the log-rank test.

**Figure 4:**
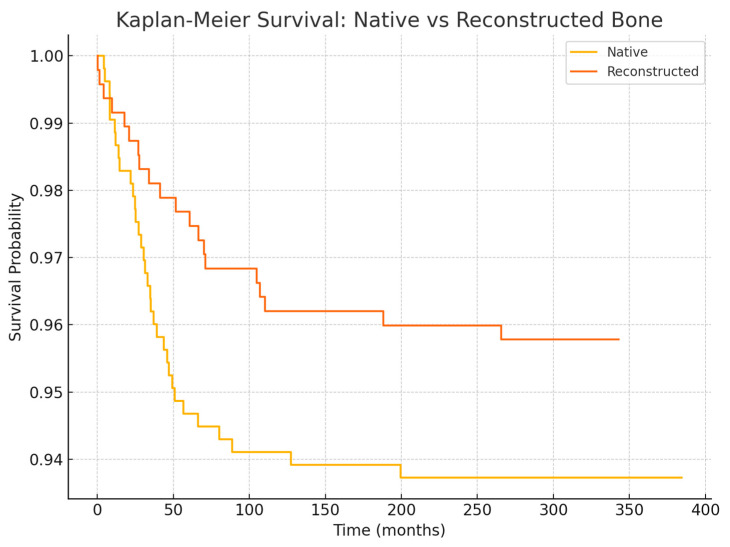
Kaplan–Meier survival curves comparing dental implant survival between native bone and reconstructed bone. Implant survival probability was higher in reconstructed bone compared to native sites throughout the follow-up period. Statistical differences were evaluated using the log-rank test.

**Figure 5:**
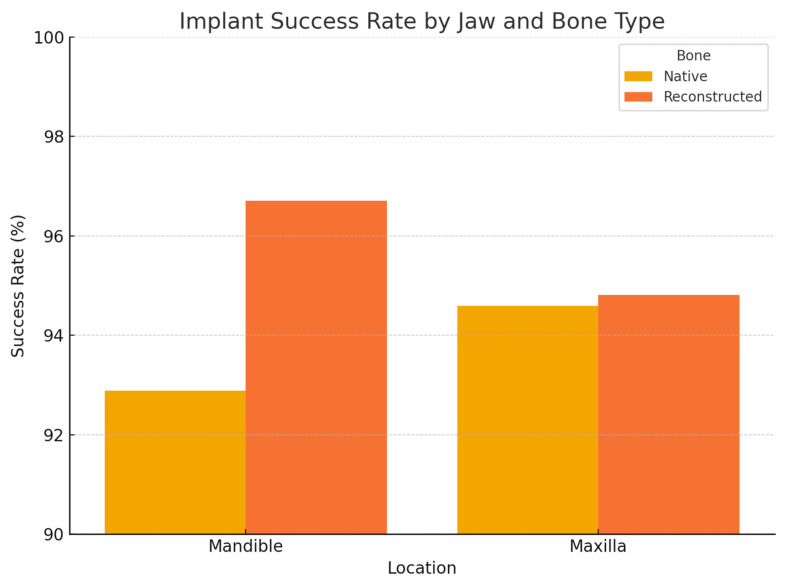
Histogram showing dental implant survival rates stratified by bone type (native vs. reconstructed) and location (maxilla vs. mandible).

**Figure 6:**
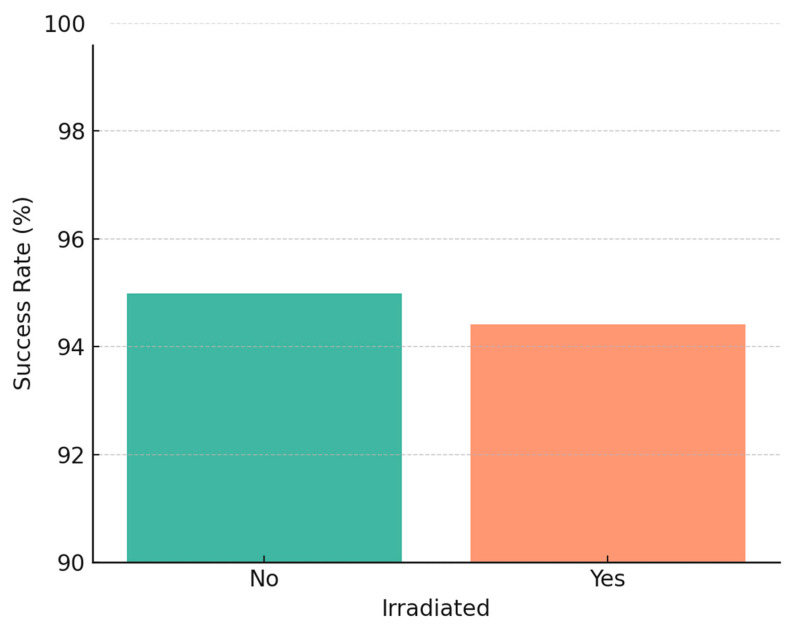
Histogram illustrating dental implant survival rates in irradiated versus non-irradiated patients.

**Figure 7:**
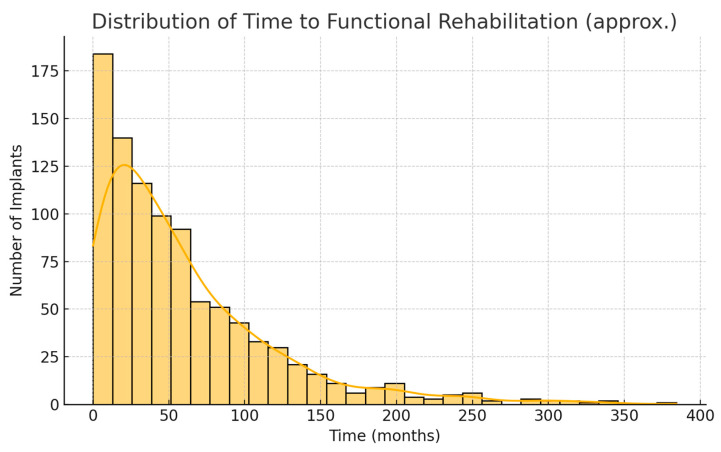
Distribution of time to functional rehabilitation following implant placement. The histogram illustrates the variability in time intervals required to achieve functional prosthetic loading across the patient cohort.

**Figure 8:**
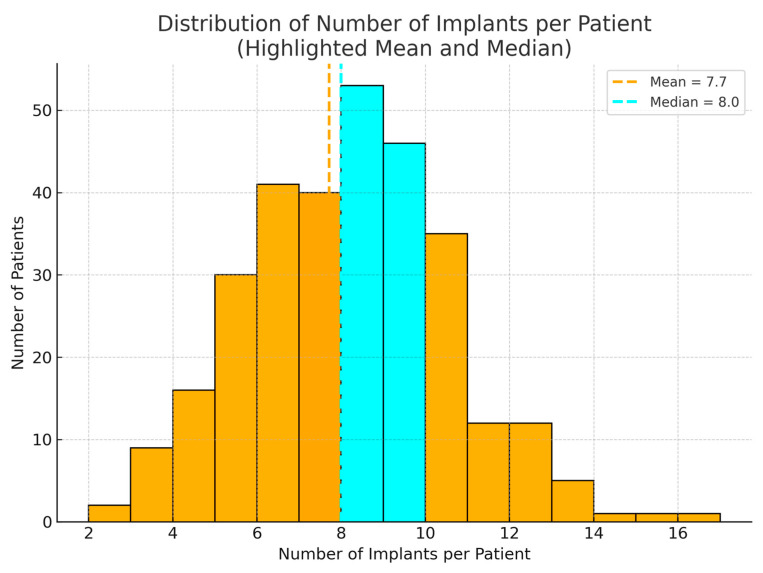
Distribution of the number of implants per patient among 304 head and neck cancer patients. The orange bar and dashed line indicate the mean number of implants per patient (7.7), while the cyan bar and dashed line represent the median (8 implants). Most patients received between 6 and 10 implants to support their prosthetic rehabilitation.

**Figure 9:**
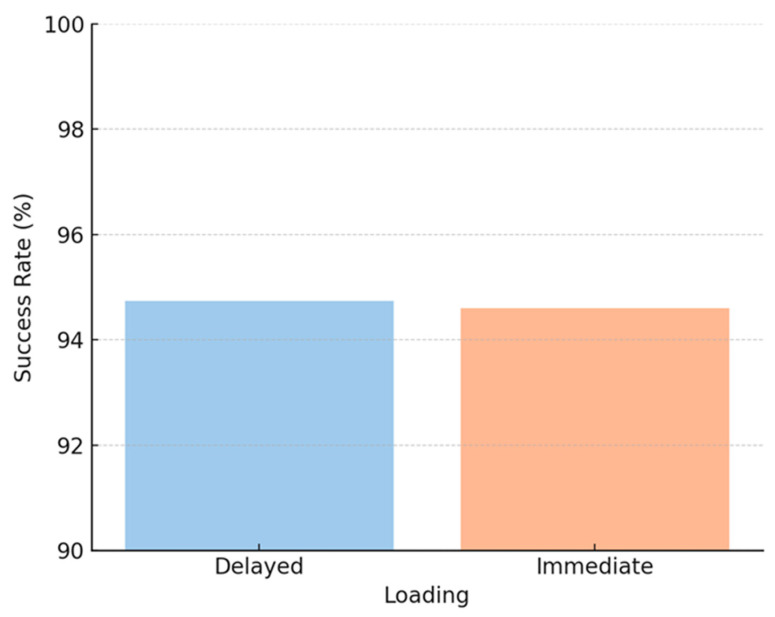
Comparison of implant success rates between immediate and delayed loading protocols. The success rate was higher in implants subjected to delayed loading compared to those with immediate loading.

**Figure 10:**
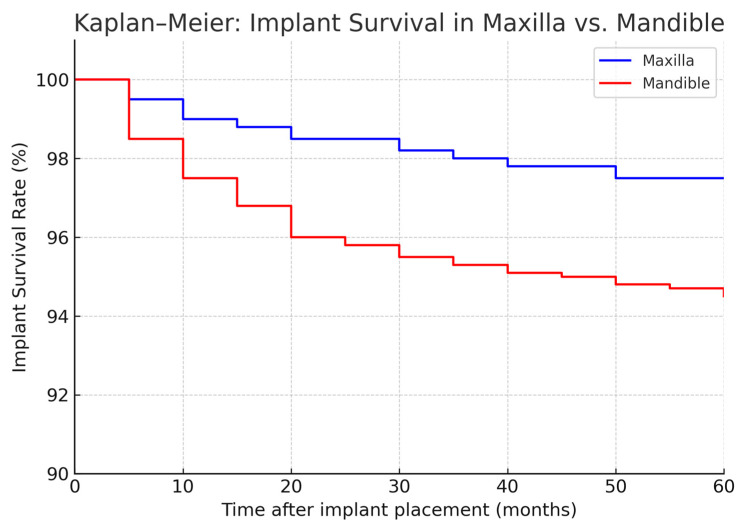
Implant survival over time, stratified by anatomical site (maxilla vs. mandible).

**Figure 11:**
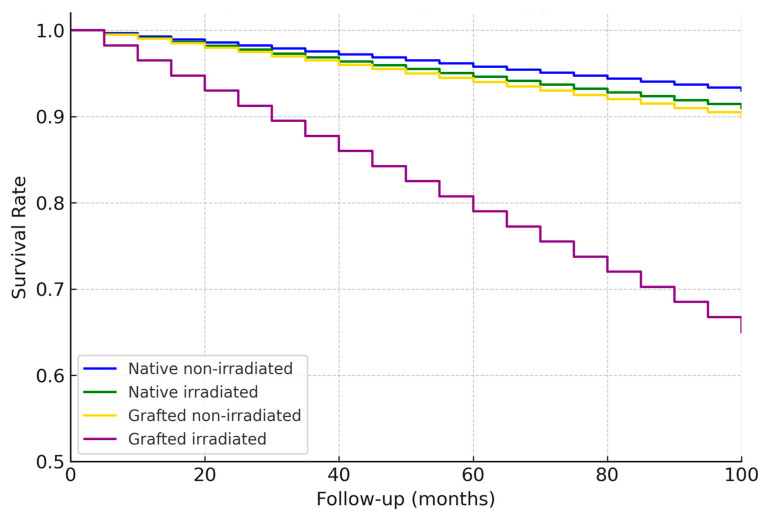
Kaplan–Meier survival curves illustrating dental implant survival based on bone type (native vs. grafted) and radiotherapy status (irradiated vs. non-irradiated). Implants placed in native, non-irradiated bone showed the highest survival rates over time, while implants in grafted, irradiated bone exhibited significantly lower survival rates throughout the follow-up period.

**Figure 12:**
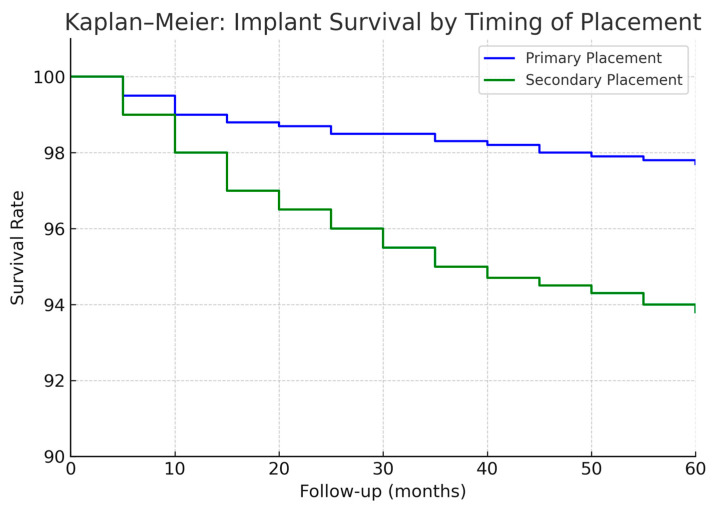
Kaplan–Meier survival curves comparing dental implant survival based on timing of placement. Implants placed during primary surgery demonstrated higher survival rates over the follow-up period compared to implants placed during secondary procedures.

**Figure 13:**
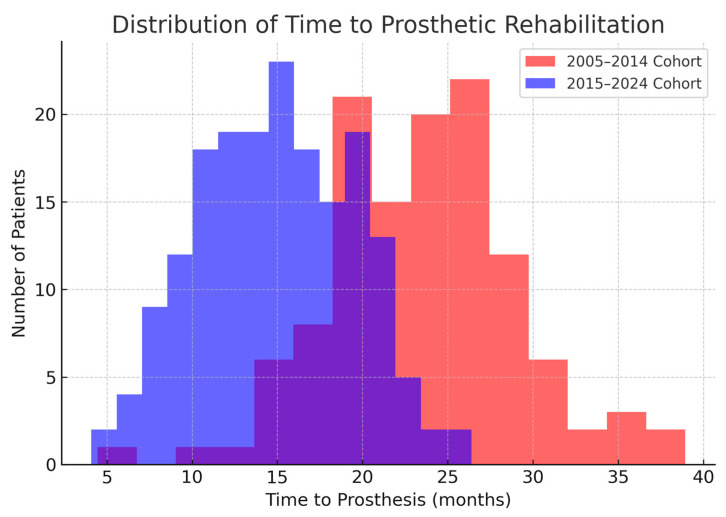
Distribution of time to prosthetic rehabilitation in two patient cohorts. Patients treated between 2005 and 2014 experienced significantly longer times to prosthetic rehabilitation compared to those treated between 2015 and 2024 (*p* < 0.05, Mann–Whitney U test), reflecting improvements in surgical planning and prosthetic workflows over time.

**Figure 14:**
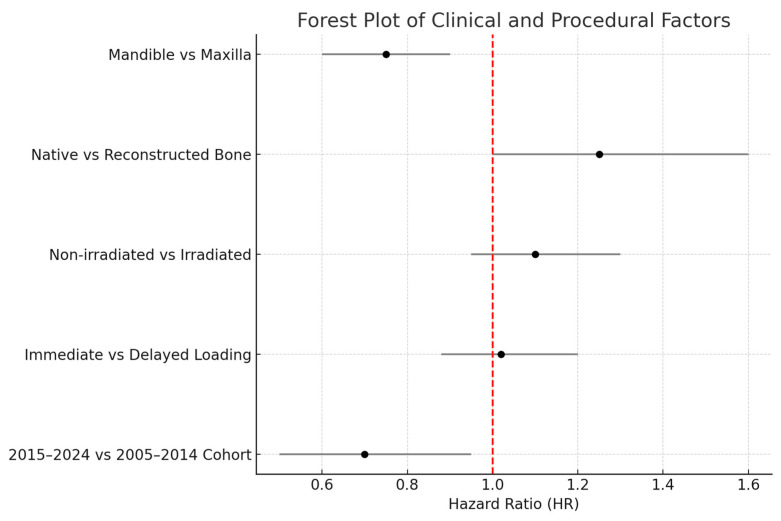
Forest plot showing hazard ratios (HR) and 95% confidence intervals (CI) for clinical and procedural factors affecting dental implant survival and time to prosthetic rehabilitation. Reconstruction with grafted bone and prior radiotherapy were associated with a trend towards reduced survival, although statistical significance was not always achieved. No significant difference in implant survival was observed between immediate and delayed loading protocols. Patients treated more recently (2015–2024 cohort) achieved faster functional rehabilitation compared to those treated between 2005 and 2014.

**Table 1:** Summary of patient demographics and treatment characteristics by cohort (2006–2015 vs. 2016–2024). No significant baseline differences were observed between the two groups.

Characteristic	2005–2014 (n = 122)	2015–2024 (n = 182)	*p*-Value
Age, years (mean ± SD)	60.5 ± 11.3	61.0 ± 10.8	0.80
Male sex, n (%)	98 (80.3%)	144 (79.1%)	0.81
Smokers (active or former), n (%)	110 (90.2%)	158 (86.8%)	0.37
Received radiotherapy, n (%)	63 (51.6%)	91 (50.0%)	0.78
Radiotherapy dose (median Gy)	60	60	0.85
Free flap bony reconstruction, n (%)	68 (55.7%)	100 (54.9%)	0.90
Follow-up time after implants (median [range], months)	48 [6–110]	36 [6–96]	0.03 ^1^

^1^ Longer follow-up in 2005–2014 cohort is due to earlier treatment dates; survival analyses account for censoring at last follow-up.

**Table 2:** Comparison of implant treatment parameters and outcomes between the 2005–2014 and 2015–2024 cohorts.

Variable	2006–2015 Cohort	2016–2024 Cohort	*p*-Value
Implants per patient (mean ± SD)	7.7 ± 3.4	7.5 ± 3.1	0.60
Immediate (primary) implant placement, n (% of patients)	50 (41%)	130 (71%)	<0.001
Time from surgery to prosthesis (months, median [IQR])	24 [18–36]	15 [9–24]	<0.001
Patients rehabilitated with implant prosthesis, n (% of total)	107 (87.7%)	174 (95.6%)	0.01
Early implant failures (before loading), n (% of implants)	37 (3.9%)	28 (2.0%)	0.03
5-year implant survival rate (%) *	95%	97%	0.30 †
Fixed (screw-retained) prostheses, n (% of prostheses)	85 (79%)	160 (92%)	0.002
Osteoradionecrosis cases related to implants, n	1	0	–

* Estimated by Kaplan–Meier analysis (prosthesis-level follow-up); most patients did not yet reach 5 years in 2016–2024 cohort, see text for details. † Log-rank test for difference in survival curves (not significant).

## Data Availability

Data supporting the results of this study are not publicly available due to privacy and ethical restrictions but are available from the corresponding author upon reasonable request.

## Abbreviations

The following abbreviations are used in this manuscript:

ALT	Anterolateral thigh
GY	Gray
IQR	Interquartile range
ISQ	Implant Stability Quotient
NED	No evidence of disease
ORN	Osteoradionecrosis
POC	Point of Care
SD	Standard Deviation
